# Elementary cellular automata realized by stateful three-memristor logic operations

**DOI:** 10.1038/s41598-024-53125-w

**Published:** 2024-02-01

**Authors:** Hongzhe Wang, Junjie Wang, Shiqin Yan, Ruicheng Pan, Mingyuan Sun, Qi Yu, Tupei Chen, Lei Chen, Yang Liu

**Affiliations:** 1 State Key Laboratory of Electronic Thin Films and Integrated Devices, University of Electronic Science and Technology of China, Chengdu, 610054 China; 2 China Changfeng Mechanics and Electronics Technology Academy, Beijing, 100039 China; 3 School of Electrical and Electronic Engineering, Nanyang Technological University, Singapore, 639798 Singapore; 4 Beijing Microelectronics Technology Institute, Beijing, 100076 China

**Keywords:** Electronic devices, Electrical and electronic engineering, Computational science

## Abstract

Cellular automata (CA) are computational systems that exhibit complex global behavior arising from simple local rules, making them a fascinating candidate for various research areas. However, challenges such as limited flexibility and efficiency on conventional hardware platforms still exist. In this study, we propose a memristor-based circuit for implementing elementary cellular automata (ECA) by extending the stateful three-memristor logic operations derived from material implication (IMP) logic gates. By leveraging the inherent physical properties of memristors, this approach offers simplicity, minimal operational steps, and high flexibility in implementing ECA rules by adjusting the circuit parameters. The mathematical principles governing circuit parameters are analyzed, and the evolution of multiple ECA rules is successfully demonstrated, showcasing the robustness in handling the stochastic nature of memristors. This approach provides a hardware solution for ECA implementation and opens up new research opportunities in the hardware implementation of CA.

## Introduction

Cellular Automata (CA) are discrete computational systems that consisting of cells on a grid. They exhibit complex global behavior arising from the simple local rules. CA was originally introduced by John von Neumann and Stanislaw Ulam in the 1940s^1^. It has been proved advantageous to various areas, e.g. cryptography^2,3^, biological modeling^4^, theoretical physics^5^, chemistry engineering^6^ and image processing^7^. In recent decades, there has been a growing interest in implementing cellular automata on hardware platforms to achieve acceleration and improve efficiency. Several studies implemented the CA on conventional hardware platforms such as FPGA^8,9^, CPU^10^ and GPU^10,11^. While some breakthroughs have been achieved, challenges like higher hardware costs and limited flexibility in evolving rules continue to be obstacles in implementing CA on conventional hardware platforms. Therefore, the exploration of non-conventional hardware platforms that are better suited for implementing CA is highly desired.

In recent years, memristor-based hardware platforms have been gaining significant prominence. Memristor is a non-linear two-terminal device whose resistance can be programmed by the applied current or voltage. It was first theorized by Leon Chua back in 1971^12^ and later experimentally confirmed by Dmitri B. Strukov et al. in 2008^13^. For over a decade, memristor-based hardware platforms has demonstrated promising properties in various areas, e.g. artificial neural networks^14,15^, in-memory computing^16,17^ and memristor-based logic^18^. One outstanding approach to realize memristor-based logic was material implication (IMP) logic gates. This innovative concept was initially introduced by Julien Borghetti et al. in 2008^19^. Building upon this idea, Ahmad Karimi et al. proposed a novel structure based on IMP logic gates in 2018^20^. Expanding on the idea of IMP logic gates, Kyung Min Kim et al.^21^ and A. Siemon et al.^22^ conducted studies on a stateful three-memristor logic gate in 2019.

In this work, we propose a memristor-based circuit design for the implementation of elementary cellular automata (ECA) by extending stateful three-memristor logic operations derived from IMP logic gates. This approach leverages the inherent physical properties of memristors, providing significant flexibility and simplicity in implementing ECA rules ranging from 0 to 255 by adjusting the circuit parameters. Through detailed circuit analysis, we determined mathematical principles governing circuit parameters and demonstrated the electrical characteristics of memristors in ECA evolution. We successfully achieved ECA evolution of various rules using the proposed memristor-based circuit design. In this approach, the memristors serve as both memory units and computing units, which effectively aligns with the concept of in-memory computing. This makes the memristor-based ECA circuit distinct from the conventional hardware platforms.

## Methods

**Figure 1:**
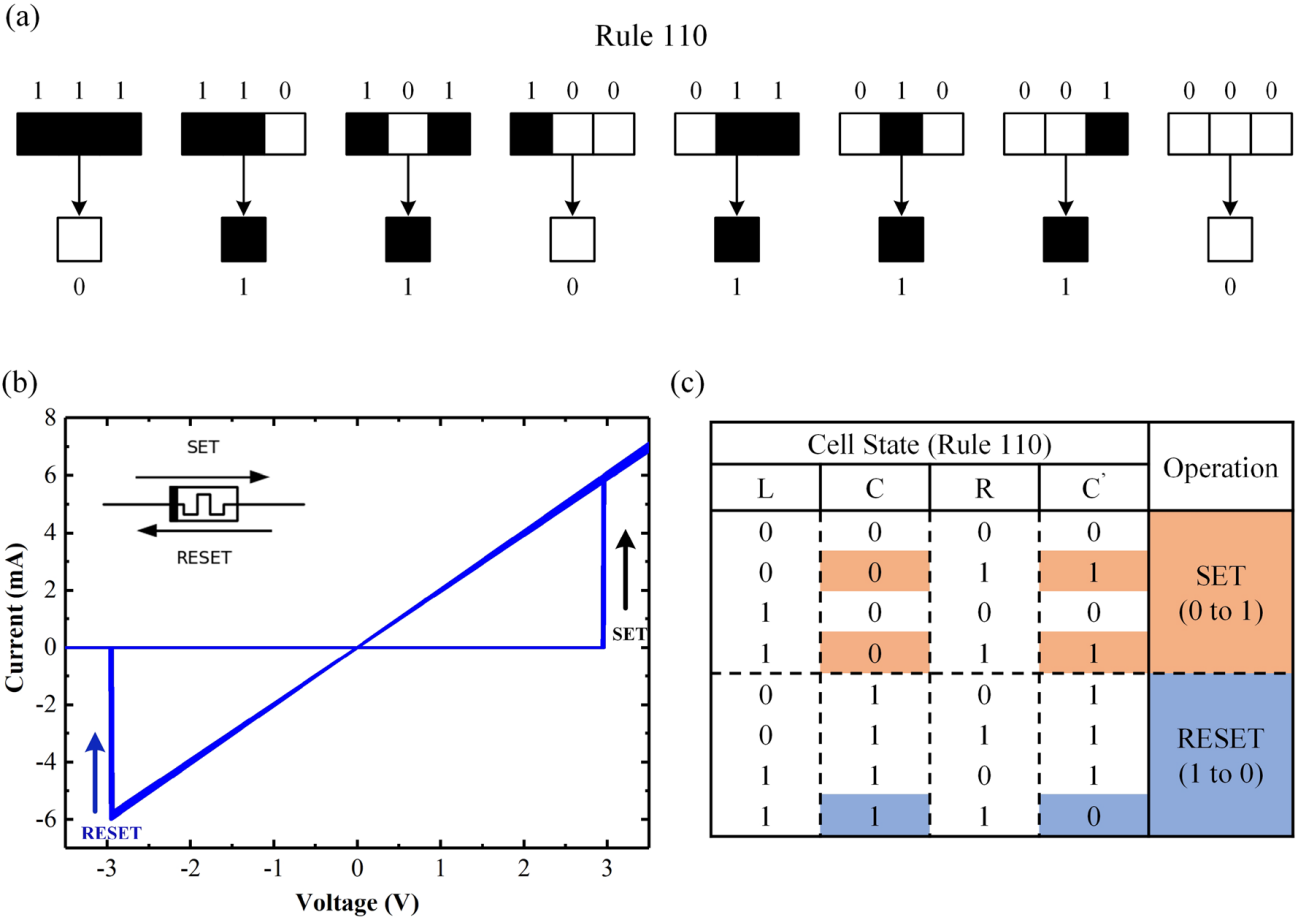
(**a**) Elementary cellular Automata, (**b**) typical hard switching characteristics of the memristor, and (**c**) logic operations divided into two stages: SET and RESET.

Elementary cellular automata (ECA) are one-dimensional cellular automata characterized by cells that can exist in two states: “0” or “1”. In ECA, the state of a cell in the next generation is determined by the current states of both the cell and the cell’s two adjacent cells. There are $2^3 = 8$ possible patterns for a cell and its two neighbors ($P_0=000$, $P_1=001$, ..., $P_6=110$, $P_7=111$). The dimension of ECA is wrapped around, hence the left neighbor of the leftmost cell is the rightmost cell, and the right neighbor or the rightmost cell is the leftmost cell. Figure 1a shows the evolutionary process of ECA governed by rule 110. This rule serves as the evolution principle, dictating the state transitions of each cell in the subsequent generation. The rule number is interpreted as a binary representation, such as 110 (in decimal), which translates to 01101110 in binary. Each digit determines the state of the cell in the next generation. For instance, the $i_{th}$ digit (counting from right to left) determines the state of the $i_{th}$ cell (counting from right to left) in the next generation. There are $2^{23}=256$ possible rules of ECA (from 0 to 255).

Figure 1b illustrates the hard-switching characteristics of the memristor model employed in this work, which is adapted from the mean metastable switch (MMSS) memristor model proposed by Knowm Inc^23^ and closely resemble the switching behaviors of a memristor like the Au/$\rm HfO_2$/Ni memristor reported in our previous study^24^. Further details about the model are described in Supplementary Note 1. In logic operations based on memristors, the low-resistance state (LRS) is defined as logical “1”, while the high-resistance state (HRS) is defined as logical “0”. To align with the characteristics of the memristor, the evolution of the ECA can be categorized into two groups of logic operations: SET and RESET, as depicted in Fig.  1c. The SET operation corresponds to the initial state of “0”, while the RESET operation corresponds to the initial state of “1”.

**Figure 2:**
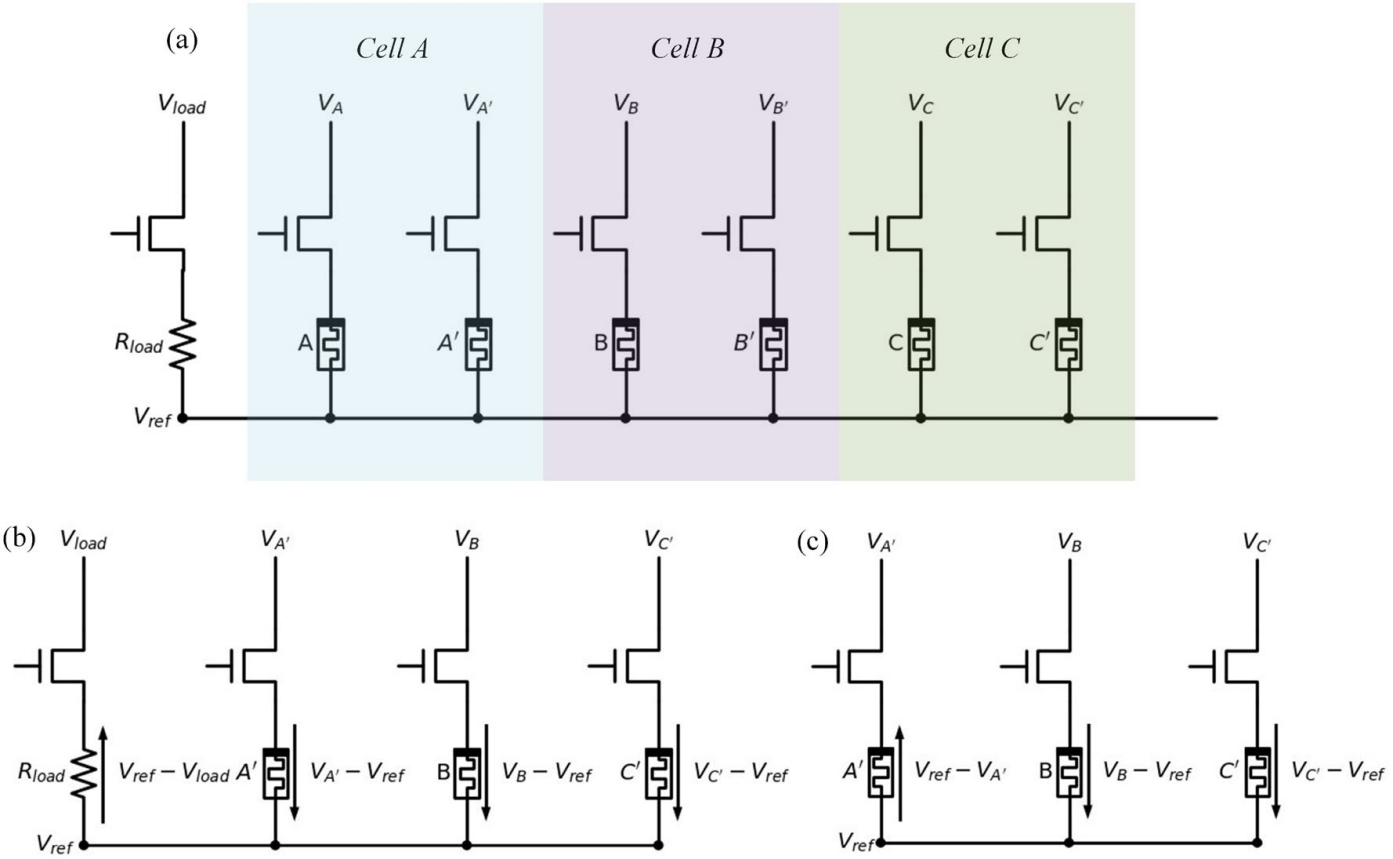
(**a**) Schematic illustration of the memristor-based ECA, (**b**) “non-floating $R_{load}$” strategy, and (**c**) “floating $R_{load}$” strategy.

Figure 2a shows the circuit schematic of the memristor-based ECA. Each cell consists of two parallel memristors connected in parallel with its neighboring cells, with one being the main memristor and the other being the dummy memristor. All cells are interconnected at their lower potential node, and a load resistor $R_{load}$ is connected from this node to ground. The resistance of $R_{load}$ is chosen to be equal to the LRS of the memristor. For clarity and easy reference, we have designated three specific cells as *A*, *B* and *C*. Each cell consists of two parallel memristors. The parallel memristors of the three cells are labeled as *A* and $A'$, *B* and $B'$, and *C* and $C'$, respectively. The memristor *A*, *B* and *C* are defined as the main memristors, while the memristor $A'$, $B'$ and $C'$ are defined as the dummy memristors in each cell. The voltages applied to the top-electrode of each memristor are defined as $V_A$, $V_{A'}$, $V_B$, $V_{B'}$, $V_C$, and $V_{C'}$, respectively. The voltages applied to $R_{load}$ is denoted as $V_{load}$. The node voltage of the common line connected to the bottom-electrode of each memristor is defined as $V_{ref}$. In the circuit, all switches (i.e., the MOSFETs shown in Fig.  2) are controlled by external signals. Assuming cell *B* is the current cell, its neighbors are cell *A* (on the left) and *C* (on the right). When voltage $V_B$ is applied, the potential difference across the memristor *B* should be $V_B - V_{ref}$. The main memristors and dummy memristors store the state of the cell for different purposes. The new state of cell *B* for the next generation is determined by the relationships among the parameters $R_{A'}$, $R_B$, $R_{C'}$, $R_{load}$, $V_{A'}$, $V_B$, $V_{C'}$, and $V_{load}$, respectively. By appropriately adjusting the voltages, the specified logic operation can be realized. To ensure the completeness of the ECA algorithm, two logic operation strategies were introduced: the “non-floating $R_{load}$” strategy and the “floating $R_{load}$” strategy, as shown in Fig. 2b,c, respectively. The rules corresponding to the “non-floating” strategy and “floating” strategy are detailed in Supplementary Note 2. The circuit underwent thorough analysis to derive the mathematical principles governing the interactions between the parameters.

Firstly, the “non-floating $R_{load}$” strategy is analyzed. When the state of cell B is “0”, the SET operation is performed. During this process, the relationships among the parameters $R_{A'}$, $R_B$, $R_{C'}$, $R_{load}$, $V_{A'}$, $V_B$, $V_{C'}$ and $V_{load}$ are obtained using Kirchhoff’s Circuit Laws, as shown in Eq. (1).

1 $$\begin{aligned} \begin{aligned} V_{SET} = {\left\{ \begin{array}{ll} \frac{R_{HRS}(V_B-V_{load})+R_{load}(2V_B-V_{A'}-V_{C'})}{R_{HRS}+3R_{load}},\;ABC \sim (000)\\ \frac{R_{HRS}R_{LRS}(V_B-V_{load})+R_{HRS}R_{load}(V_B-V_{C'})+R_{LRS}R_{load}(V_B-V_{A'})}{R_{HRS}R_{LRS}+R_{HRS}R_{load}+2R_{LRS}R_{load}},\;ABC \sim (001)\\ \frac{R_{HRS}R_{LRS}(V_B-V_{load})+R_{HRS}R_{load}(V_B-V_{A'})+R_{LRS}R_{load}(V_B-V_{C'})}{R_{HRS}R_{LRS}+R_{HRS}R_{load}+2R_{LRS}R_{load}},\;ABC \sim (100)\\ \frac{R_{HRS}[R_{LRS}(V_B-V_{load})+R_{load}(2V_B-V_{A'}-V_{C'})]}{R_{HRS}R_{LRS}+2R_{HRS}R_{load}+R_{LRS}R_{load}},\;ABC \sim (101) \end{array}\right. } \end{aligned} . \end{aligned}$$

The value of $R_{load}$ is much smaller than $R_{HRS}$, which can be considered negligible in the above equation when $ABC \sim (000)$ (i.e., the cell state of cell *A*, *B* and *C* is “0”, “0” and “0”, respectively). Similarly, the value of $R_{LRS}R_{load}$ is much smaller than $R_{LRS}R_{HRS}$ and $R_{load}R_{HRS}$, which can be neglected in the equation when $ABC \sim (001), (100)\,\rm or \,(101)$. Therefore, Eq. (1) can be simplified as Eq. (2).

2 $$\begin{aligned} V_{SET} = {\left\{ \begin{array}{ll} V_B-V_{load}, ABC \sim (000)\\ \frac{R_{LRS}(V_B-V_{load})+R_{load}(V_B-V_{C'})}{R_{LRS}+R_{load}},\;ABC \sim (001)\\ \frac{R_{LRS}(V_B-V_{load})+R_{load}(V_B-V_{A'})}{R_{LRS}+R_{load}},\;ABC \sim (100)\\ \frac{R_{LRS}(V_B-V_{load})+R_{load}(2V_B-V_{A'}-V_{C'})}{R_{LRS}+2R_{load}},\;ABC \sim (101) \end{array}\right. } . \end{aligned}$$

The values of $V_{SET}^{(000)}$, $V_{SET}^{(001)}$, $V_{SET}^{(100)}$ and $V_{SET}^{(101)}$ depend on $V_{A'}$, $V_B$, $V_{C'}$ and $V_{load}$. If $V_{SET}^{(ABC)}$ is greater than the SET threshold of memristor, the state of cell B changes from “0” to “1”. Otherwise, the state of cell B remains “0”. By applying appropriate $V_{A'}$, $V_B$, $V_{C'}$ and $V_{load}$, the conditional SET operation with the “non-floating $R_{load}$” strategy can be realized.

To ensure the completeness of the ECA rules from 0 to 255, it is necessary to employ the “floating $R_{load}$” strategy during the SET stage. The relationships among the parameters $R_{A'}$, $R_B$, $R_{C'}$, $V_{A'}$, $V_B$ and $V_{C'}$ can be obtained from the Kirchhoff Circuit Laws, as shown in Eq. (3).

3 $$\begin{aligned} V_{SET} = {\left\{ \begin{array}{ll} \frac{2V_B-V_{A'}-V_{C'}}{3},\;ABC \sim (000)\\ \frac{R_{HRS}(V_B-V_{C'})+R_{LRS}(V_B-V_{A'})}{R_{HRS}+2R_{LRS}},\;ABC \sim (001)\\ \frac{R_{HRS}(V_B-V_{A'})+R_{LRS}(V_B-V_{C'})}{R_{HRS}+2R_{LRS}},\;ABC \sim (100)\\ \frac{R_{HRS}(2V_B-V_{A'}-V_{C'})}{2R_{HRS}+R_{LRS}},\;ABC \sim (101) \end{array}\right. } . \end{aligned}$$

As the value of $R_{LRS}$ is much smaller than $R_{HRS}$, which can be considered negligible in the above equation when $ABC \sim (001), (100)\,\rm or \,(101)$. Therefore, Eq. (3) can be simplified as Eq. (4),

4 $$\begin{aligned} V_{SET} = {\left\{ \begin{array}{ll} \frac{2V_B-V_{A'}-V_{C'}}{3},\;ABC \sim (000)\\ V_B-V_{C'},\;ABC \sim (001)\\ V_B-V_{A'},\;ABC \sim (100)\\ \frac{2V_B-V_{A'}-V_{C'}}{2},\;ABC \sim (101) \end{array}\right. } . \end{aligned}$$

The values of $V_{SET}^{(000)}$, $V_{SET}^{(001)}$, $V_{SET}^{(100)}$ and $V_{SET}^{(101)}$ depend on $V_{A'}$, $V_B$ and $V_{C'}$. If $V_{SET}^{(ABC)}$ is greater than the SET threshold of the memristor, the state of cell B changes from “0” to “1”. Otherwise, the state of cell B remains “0”. By applying appropriate $V_{A'}$, $V_B$ and $V_{C'}$, conditional SET operation with the “floating $R_{load}$” strategy can be realized.

When the state of cell B is “1”, the RESET operation is performed. For this operation, the relationships among the parameters $R_{A'}$, $R_B$, $R_{C'}$, $R_{load}$, $V_{A'}$, $V_B$, $V_{C'}$ and $V_{load}$ are obtained from Kirchhoff’s Circuit Laws, as shown in Eq. (5).

5 $$\begin{aligned} V_{RESET} = {\left\{ \begin{array}{ll} \frac{R_{LRS}[R_{HRS}(V_B-V_{load})+R_{load}(2V_B-V_{A'}-V_{C'})]}{R_{HRS}R_{LRS}+2R_{LRS}R_{load}+R_{HRS}R_{load}},\;ABC \sim (010)\\ \frac{R_{HRS}R_{LRS}(V_B-V_{load})+R_{HRS}R_{load}(V_B-V_{C'})+R_{LRS}R_{load}(V_B-V_{A'})}{R_{HRS}R_{LRS}+2R_{HRS}R_{load}+R_{LRS}R_{load}},\;ABC \sim (011)\\ \frac{R_{HRS}R_{LRS}(V_B-V_{load})+R_{HRS}R_{load}(V_B-V_{A'})+R_{LRS}R_{load}(V_B-V_{C'})}{R_{HRS}R_{LRS}+2R_{HRS}R_{load}+R_{LRS}R_{load}},\;ABC \sim (110)\\ \frac{R_{LRS}(V_B-V_{load})+R_{load}(2V_B-V_{A'}-V_{C'})]}{R_{LRS}+3R_{load}},\;ABC \sim (111) \end{array}\right. } . \end{aligned}$$

The value of $R_{LRS}R_{load}$ is much smaller than $R_{LRS}R_{HRS}$ and $R_{load}R_{HRS}$, which can be considered negligible in the above equation when $ABC \sim (010), (011)\,\rm or \,(110)$. Therefore, Eq. (5) can be further simplified as Eq. (6).

6 $$\begin{aligned} V_{RESET} = {\left\{ \begin{array}{ll} \frac{R_{LRS}(V_B-V_{load})}{R_{LRS}+R_{load}},\;ABC \sim (010)\\ \frac{R_{LRS}(V_B-V_{load})+R_{load}(V_B-V_{C'})}{R_{LRS}+2R_{load}},\;ABC \sim (011)\\ \frac{R_{LRS}(V_B-V_{load})+R_{load}(V_B-V_{A'})}{R_{LRS}+2R_{load}},\;ABC \sim (110)\\ \frac{R_{LRS}(V_B-V_{load})+R_{load}(2V_B-V_{A'}-V_{C'})]}{R_{LRS}+3R_{load}},\;ABC \sim (111) \end{array}\right. } . \end{aligned}$$

The value of $V_{RESET}^{(010)}$, $V_{RESET}^{(011)}$, $V_{RESET}^{(110)}$ and $V_{RESET}^{(111)}$ are dependent on $V_{A'}$, $V_B$, $V_{C'}$ and $V_{load}$. If $V_{RESET}^{(ABC)}$ is greater than RESET threshold of memristor, state of cell B changes from “1” to “0”. Otherwise, the state of cell B remains “1”. By applying appropriate $V_{A'}$, $V_B$, $V_{C'}$ and $V_{load}$, the conditional RESET operation can be realized. Being different from the SET stage, the RESET stage of all ECA rules can be realized based on the “non-floating $R_{load}$” strategy. Therefore, the “floating $R_{load}$” strategy is not necessary for the RESET stage.

There are 16 types of conditional SET stages and 16 types of conditional RESET stages for the ECA rule, ranging from 0 to 255. Most of them can be accomplished through a single operation, except for SET operations with $P_5P_4P_1P_0=(0110)\,\rm and \,(1001)$ and RESET operations with $P_7P_6P_3P_2 = (0110)\,\rm and \,(1001)$. These specific SET/RESET stages can be achieved by performing two consecutive steps of operations.

## Results and discussion

**Figure 3:**
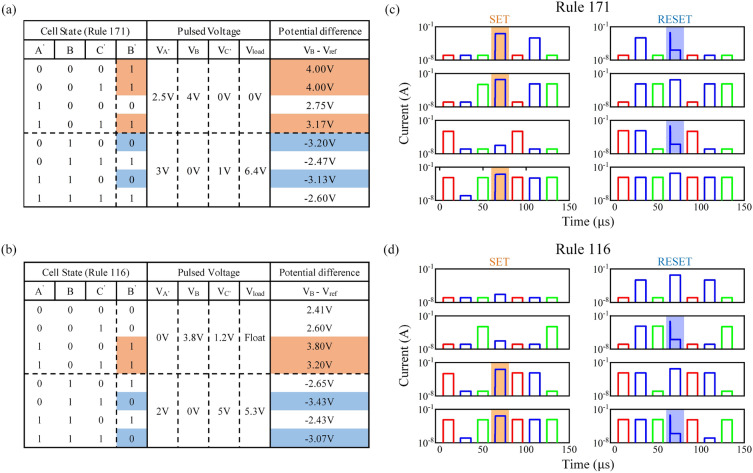
Node potentials during the evolution of memristor-based ECA with (**a**) Rule 171 and (**b**) Rule 116; The elertrical charactristic of memristor during evolution with (**c**) Rule 171 and (**d**) Rule 116.

Figure 3 demonstrates the stateful three-memristor logic operations for ECA through simulation. Circuit-level simulation is conducted using LTspice XVII, with a Python-LTspice interface developed to control the entire ECA evolution process, providing flexibility to modify rules and collecting circuit parameters. The HRS and LRS of the memristors and the load resistor were set as follows: $R_{HRS} = 5\times 10^6 \Omega$, $R_{LRS} = 5\times 10^2 \Omega$ and $R_{load} = 5\times 10^2 \Omega$; where the positive/negative threshold of memristor was defined as: $V_{SET} = 3V$, $V_{RESET} = -3V$, respectively. The node potentials during the SET stage and RESET stage for Rule 171 and Rule 116 of the memristor-based ECA are shown in Fig.  3a,b, respectively. Rule 171 employs the “non-floating” strategy; while Rule 116 employs the “floating” strategy. Figure 3c,d show the electrical characteristic of the memristor *A*, *B* and *C* during evolution, respectively. The red, blue and green line corresponds to the memristor $A'$, *B* and $C'$, respectively. Firstly, the READ operation was implemented, a voltage pulse with amplitude 0.1V and width 12 μs was sequentially applied to memristor $A'$, *B* and $C'$, for reading the current state of memristor (cell). The resistance state of memristor can be measured by the magnitude of the reading current, where the HRS ($<10\,\upmu \text{A}$) and the LRS ($\ge 10\,\upmu \text{A}$). Subsequently, the conditional SET/RESET operation was carried out, the pulses with amplitude $V_{A'}$, $V_B$ and $V_{C'}$ and width 12 μs were simultaneously applied to memristor $A'$, *B* and $C'$, respectively. After that, another READ operation was implemented to read the state of each memristor. The results of the stateful three-memristor logic operations for Rule 171 and Rule 116 are consistent with the predicted outcomes.

**Figure 4:**
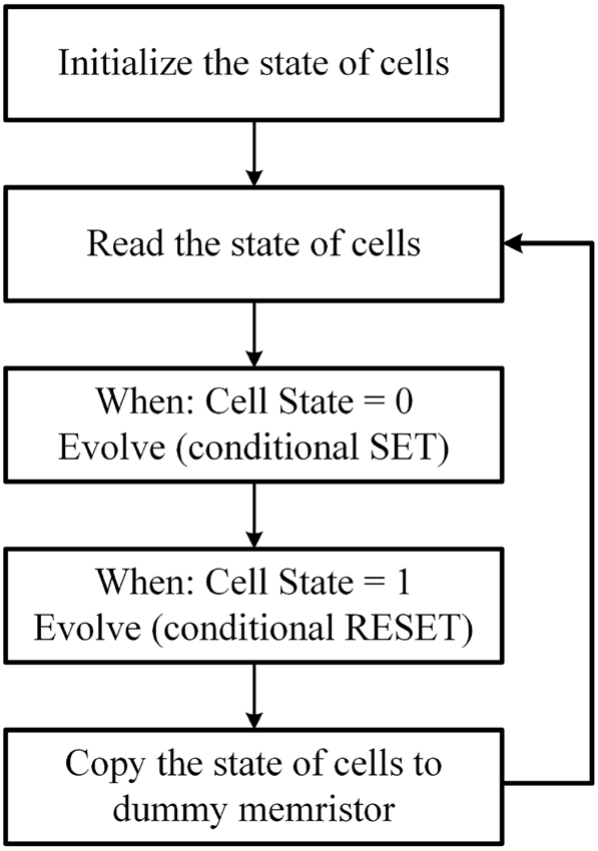
Flowchart of the memristor-based ECA evolution.

The ECA typically involves multiple cycles for evolution, requiring a comprehensive hardware control approach. Figure 4 illustrates the flowchart of the memristor-based ECA evolution. In this circuit, the initial state of all memristors is set to “0”. Therefore, the memristors in state “1” are initially RESET to the HRS. Subsequently, each cell state of the ECA is mapped to the corresponding memristors. For the cells in state “1”, the corresponding memristors are SET to the low-resistance state (LRS). Following this, a small fixed voltage pulse is applied to measure the current flowing through each memristor. The resistance state (HRS or LRS) of the memristor can be determined based on the measured current, which is then used to determine whether each cell should undergo the SET stage or RESET stage during evolution. After completing the SET stage for all cells in state “0” and the RESET stage for all cells in state “1”, dummy memristors are employed to synchronize the cell states for the next generation. The COPY operation^21^ is utilized to synchronize the states of the main memristors with their corresponding dummy memristors. Once this step is completed, another evolution cycle is implemented to continue the ECA evolution process.

**Figure 5:**
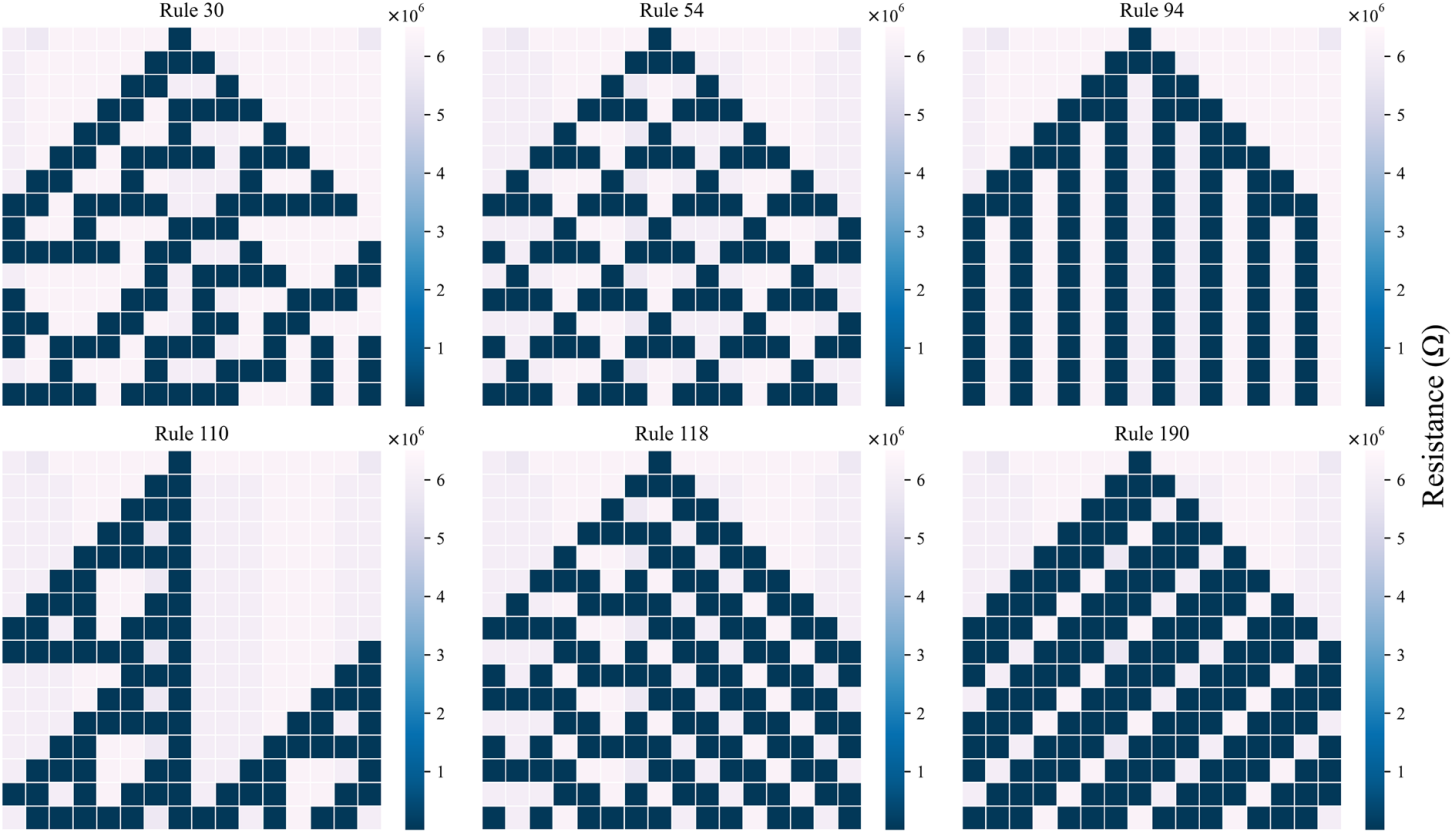
Evolution of the memristor-based ECA with Rules 30, 54, 94, 110, 118 and 190.

Figure 5 shows the evolution of the memristor-based ECA using different rules: 30, 54, 94, 110, 118 and 190. The ECA comprises 32 memristors, i.e., 16 main memristors and 16 dummy memristors, forming a total of 16 cells. The ECA’s initial state is specified as “1” in the 8th cell from the left, while the remaining cells are set to “0”. It undergoes evolution for a total of 15 cycles. The results of the evolution align with the expected outcomes for all rules, demonstrating the accuracy of the proposed memristor-based ECA. The system exhibits solid robustness even in the presence of stochastic characteristics, as evidenced by the successful evolution despite the inclusion of 10% white noise in $R_{ON}$ and $R_{OFF}$ and a 5% white noise component in the $V_{ON}$ and $V_{OFF}$ in the adapted MMSS memristor model. Additionally, the error tolerance of parameters $R_{ON}$, $R_{OFF}$, $V_{ON}$, and $V_{OFF}$ in the adapted MMSS memristor model is analyzed in Supplementary Note 3. The results of the evolution are consistent with the expected outcomes for all rules, demonstrating the accuracy and robustness of the proposed memristor-based ECA. Despite some fluctuations in the resistance of the memristors during the evolution process, they do not have an impact on the overall results.

## Conclusion

In this study, we have introduced a memristor-based ECA circuit design capable of encompassing ECA rules from 0 to 255. Memristors serve as both storage elements for cell states and computing elements of ECA evolution by applying appropriate voltages to each node. The ECA evolution process is efficiently divided into two stages, SET and RESET, by leveraging the characteristics of memristors and employing stateful three-memristor logic operations. Through detailed circuitry analysis, we have successfully identified the mathematical principles that govern each parameter of the proposed memristor-based ECA. This comprehensive understanding enables us to effectively achieve the desired ECA rule. SPICE simulations were conducted to demonstrate the evolution process of the memristor-based ECA for various rules. The proposed approach successfully achieved the expected evolution patterns of the memristor-based ECA and exhibited solid robustness in handling the inherent stochasticity of memristors. With its simplicity, minimal operational steps and high flexibility, the proposed approach opens an avenues for further research in the hardware implementation of CA.

### Supplementary Information

Supplementary Information.

## Data Availability

The datasets generated during and/or analysed during the current study are available from the corresponding author on reasonable request.
