# Supplementary material for: Elementary cellular automata realized by stateful three-memristor logic operations
Source: Sci Rep. 2024 Feb 1;14:2677. doi: 10.1038/s41598-024-53125-w (PMC10834433; doi:10.1038/s41598-024-53125-w)
Supplement: Supplementary file 1 — Supplementary Information. [file 41598_2024_53125_MOESM1_ESM.docx]

**Elementary Cellular Automata realized by stateful three-memristor logic operations**

Hongzhe Wang^1^, Junjie Wang^1,*^, Shiqin Yan^1^, Ruicheng Pan^1^, Mingyuan Sun^2^, Qi Yu^1^, Tupei Chen^3^, Lei Chen^4^ and Yang Liu^1^

^1^State Key Laboratory of Electronic Thin Films and Integrated Devices, University of Electronic Science and Technology of China, Chengdu 610054, China

^2^China Changfeng Mechanics and Electronics Technology Academy, Beijing 100039, China

^3^School of Electrical and Electronic Engineering, Nanyang Technological University, Singapore 639798, Singapore

^4^Beijing Microelectronics Technology Institute, Beijing 100076, China

**Supplementary Note 1: The adapted mean metastable switch (MMSS) memristor model**

$G\left( X \right)=\frac{S\left( x \right)}{R_{ON}}+\frac{S\left( 1-X \right)}{R_{OFF}}$ (S1)

$S\left( X \right)=\frac{1}{1+\left( \frac{X}{1-X} \right)^{\alpha}}$ (S2)

$\frac{dX}{dt}= \frac{1}{\tau}\left[ \frac{1}{1+e^{-\beta\left( V-V_{ON} \right)}}\cdot\left( 1-X \right)-(1-\frac{1}{1+e^{-\beta\left( V+V_{OFF} \right)}})\cdot X \right]$ (S3)

In Eq. S1, $G\left( x \right)$ represents the conductance of a memristor. The state variable $X$ of the memristor has a value between 0 and 1. When $X$= 0 or 1, $G\left( x \right)$ reaches its minimum or maximum value, i.e., the memristor reaches its maximum resistance $R_{OFF}$ or minimum resistance $R_{ON}$, respectively. The adapted MMSS memristor model employs an S-shaped curve function, denoted as $S\left( X \right)$, to closely resemble the characteristics of hard-switching. In Eq. S2, $\alpha(<0)$ is a tunable constant which is used to adjust nonlinearity of $S\left( X \right)$ function ($it enables S\left( 0.5 \right)=0.5, \lim_{X\to0} S\left( X \right)=0, \underset{X\to1}{and lim} S\left( X \right)=1.$). In Eq. S3, $\frac{dX}{dt}$ represents the drift velocity of the state variable; $\beta=\frac{q}{kT}= \left( V_{T} \right)^{-1}$ where $V_{T}$ is the thermal voltage, $q$ is the elementary charge, $k$ is the Boltzmann constant, and $T$ is the absolute temperature of the device; $V_{ON}$ and $V_{OFF}$ represent the positive and negative threshold voltage of the memristor, respectively; and $\tau$ is a time constant. Table S1 shows the parameters of the adapted MMSS memristor model utilized in this study.

**Table S1 The parameters of the adapted MMSS memristor model.**

| *R_ON_* | *R_OFF_* | *V_ON_* | *V_OFF_* | $\alpha$ | $\tau$ | *T* |
| --- | --- | --- | --- | --- | --- | --- |
| 5×10^2^ | 5×10^6^ | 3 | 3 | -500 | 1×10^-9^ | 298.5 |

**Supplementary Note 2: "non-floating" and "floating" strategy for the SET operation**

Table S2 shows the ECA rules realized by the "non-floating" strategy, comprising a total of 192 ECA rules. Meanwhile, Table S3 shows the rules realized by the "floating" strategy, comprising a total of 32 ECA rules. Additionally, for the 16 rules corresponding to *P_5_P_4_P_1_P_0_* = (0000), no SET operation is required. For the 16 rules corresponding to *P_5_P_4_P_1_P_0_* = (1111), SET operation is simply applied without consideration for neighboring cells.

**Table S2 ECA rules requires "non-floating" strategy for the SET operation.**

| P_5_P_4_P_1_P_0_ | ECA Rule | | | | | | | |
| --- | --- | --- | --- | --- | --- | --- | --- | --- |
| 0001 | 1 | 5 | 9 | 13 | 65 | 69 | 73 | 77 |
|  | 129 | 133 | 137 | 141 | 193 | 197 | 201 | 205 |
| 0010 | 2 | 6 | 10 | 14 | 66 | 70 | 74 | 78 |
|  | 130 | 134 | 138 | 142 | 194 | 198 | 202 | 206 |
| 0011 | 3 | 7 | 11 | 15 | 67 | 71 | 75 | 79 |
|  | 131 | 135 | 139 | 143 | 195 | 199 | 203 | 207 |
| 0100 | 16 | 20 | 24 | 28 | 80 | 84 | 88 | 92 |
|  | 144 | 148 | 152 | 156 | 208 | 212 | 216 | 220 |
| 0101 | 17 | 21 | 25 | 29 | 81 | 85 | 89 | 93 |
|  | 145 | 149 | 153 | 157 | 209 | 213 | 217 | 221 |
| 0110 | 18 | 22 | 26 | 30 | 82 | 86 | 90 | 94 |
|  | 146 | 150 | 154 | 158 | 210 | 214 | 218 | 222 |
| 0111 | 19 | 23 | 27 | 31 | 83 | 87 | 91 | 95 |
|  | 147 | 151 | 155 | 159 | 211 | 215 | 219 | 223 |
| 1000 | 32 | 36 | 40 | 44 | 96 | 100 | 104 | 108 |
|  | 160 | 164 | 168 | 172 | 224 | 228 | 232 | 236 |
| 1001 | 33 | 37 | 41 | 45 | 97 | 101 | 105 | 109 |
|  | 161 | 165 | 169 | 173 | 225 | 229 | 233 | 237 |
| 1011 | 35 | 39 | 43 | 47 | 99 | 103 | 107 | 111 |
|  | 163 | 167 | 171 | 175 | 227 | 231 | 235 | 239 |
| 1101 | 49 | 53 | 57 | 61 | 113 | 117 | 121 | 125 |
|  | 177 | 181 | 185 | 189 | 241 | 245 | 249 | 253 |
| 1110 | 50 | 54 | 58 | 62 | 114 | 118 | 122 | 126 |
|  | 178 | 182 | 186 | 190 | 242 | 246 | 250 | 254 |

**Table S3 ECA rules requires "floating" strategy for the SET operation**

| P_5_P_4_P_1_P_0_ | ECA Rule | | | | | | | |
| --- | --- | --- | --- | --- | --- | --- | --- | --- |
| 1010 | 34 | 38 | 42 | 46 | 98 | 102 | 106 | 110 |
|  | 162 | 166 | 170 | 174 | 226 | 230 | 234 | 238 |
| 1100 | 48 | 52 | 56 | 60 | 112 | 116 | 120 | 124 |
|  | 176 | 180 | 184 | 188 | 240 | 244 | 248 | 252 |

**Supplementary Note 3: Error Tolerance of the memristor-based ECA**

Table S4 illustrates the error tolerance of *R_ON_*, *R_OFF_*, *V_ON_* and *V_OFF_* in the adapted MMSS memristor model for ECA rules used in Fig. 5. Each parameter is individually subjected to variation while keeping the other parameters fixed, continuing until the ECA fails to evolve correctly. The results suggest that the robustness of memristor-based ECA is primarily influenced by variations in *V_ON_* and *V_OFF_*, followed by the deviations of *R_ON_*. The influence of *R_OFF_* on the robustness of ECA is relatively minor.

**Table S4 Error tolerance of parameters for the memristor-based ECA.**

| ECA Rule | Error tolerance (%) | | | |
| --- | --- | --- | --- | --- |
|  | *R_ON_* | *R_OFF_* | *V_ON_* | *V_OFF_* |
| 30 | 23.62% | ＞99% | 7.74% | 6.98% |
| 54 | 36.17% | ＞99% | 11.40% | 12.27% |
| 94 | 55.14% | ＞99% | 7.21% | 7.82% |
| 110 | 54.70% | ＞99% | 7.84% | 7.82% |
| 118 | 22.61% | ＞99% | 10.13% | 6.98% |
| 190 | 15.60% | ＞99% | 7.32% | 9.24% |
